# Effect of Trastuzumab–HER2 Complex Formation on Stress-Induced Modifications in the CDRs of Trastuzumab

**DOI:** 10.3389/fchem.2021.794247

**Published:** 2022-01-03

**Authors:** Baubek Spanov, Victoria Aboagye, Oladapo Olaleye, Natalia Govorukhina, Nico C. van de Merbel, Rainer Bischoff

**Affiliations:** ^1^ Department of Analytical Biochemistry, Groningen Research Institute of Pharmacy, University of Groningen, Groningen, Netherlands; ^2^ Bioanalytical Laboratory, ICON, Assen, Netherlands

**Keywords:** trastuzumab, HER2, deamidation, trastuzumab–HER2 complex, cation-exchange chromatography, size-exclusion chromatography, peptide mapping

## Abstract

Asparagine deamidation and aspartic acid isomerization in the complementarity determining regions (CDRs) of monoclonal antibodies may alter their affinity to the target antigen. Trastuzumab has two hot spots for deamidation and one position for isomerization in the CDRs. Little is known how complex formation with its target antigen HER2 affects these modifications. Modifications in the CDRs of trastuzumab were thus compared between the free antibody and the trastuzumab–HER2 complex when stressed under physiological conditions at 37°C. Complex formation and stability of the complex upon stressing were assessed by size-exclusion chromatography. Deamidation of light-chain Asn-30 (Lc-Asn-30) was extensive when trastuzumab was stressed free but reduced about 10-fold when the antibody was stressed in complex with HER2. Almost no deamidation of heavy-chain (Hc-Asn-55) was detected in the trastuzumab–HER2 complex, while deamidation was observed when the antibody was stressed alone. Hc-Asp-102 isomerization, a modification that critically affects biological activity, was observed to a moderate degree when the free antibody was stressed but was not detected at all in the trastuzumab–HER2 complex. This shows that complex formation has a major influence on critical modifications in the CDRs of trastuzumab.

## Introduction

Monoclonal antibodies (mAbs) are an important class of biopharmaceuticals for the treatment of a variety of severe diseases due to their high specificity and long half-life (Chames et al., 2009). The high specificity is defined by the antigen-binding fragment (Fab) of mAbs. The Fab consists of heavy and light chains of an antibody connected by an inter-chain disulfide bond. Each chain has three complementarity determining regions (CDRs), hypervariable loops that consist of several amino acid residues forming the antigen-binding sites. Formation of the antigen–antibody complex is governed by electrostatic and hydrophobic interactions between amino acid residues of the CDRs and epitope(s) of the target antigen (Davies et al., 1990). Chemical change of CDR amino acids both *in vitro* and *in vivo* due to susceptibility to modifications, such as asparagine deamidation or aspartic acid isomerization, may have a negative effect on antigen binding or diminish potency in cell-based assays (Cacia et al., 1996; Vlasak et al., 2009). It is hard to predict the impact of CDR modifications on antigen binding because each particular antibody represents a unique case. For example, deamidation in the heavy chain CDR2 resulted in 14 times reduction in binding affinity of a proprietary mAb (Huang et al., 2005). However, for another mAb, deamidation in the heavy chain CDR2 had no effect on potency in a cell-based assay (Lyubarskaya et al., 2006). Besides CDRs, amino acid residues of the framework regions (FR) may play an important role in the generation of high-affinity antibodies. Firstly, FR amino acids can be in contact with the antigen when the antibody–antigen complex is formed. Secondly, FR amino acids can contribute to antigen binding by affecting the conformation of a particular CDR. Humanization studies of trastuzumab showed that replacement of FR amino acids at particular positions resulted in higher affinity variants (Carter et al., 1992).

Trastuzumab is a recombinant humanized mAb that targets sub-domain IV of the extracellular domain of human epidermal growth factor receptor 2 (HER2). After Food and Drug Administration (FDA) approval in 1998, trastuzumab is presently one of the main drugs used for the treatment of HER2-positive breast cancer at different stages. Harris et al. were the first to show charge heterogeneity of trastuzumab by separating charge variants by cation-exchange chromatography (Harris et al., 2001). The source of heterogeneity was due to asparagine deamidation and aspartic acid isomerization in the CDRs of trastuzumab. Later, several other studies presented a similar charge heterogeneity profile of trastuzumab confirming that cation-exchange chromatography is a reliable approach for charge variant separation (Lingg et al., 2013; Schmid et al., 2018; Spanov et al., 2021). Some studies reported that acidic variants of trastuzumab caused by asparagine deamidation have a lower affinity to HER2 compared to the unmodified antibody isolated by cation-exchange chromatography (Dakshinamurthy et al., 2017; Schmid et al., 2018). Harris et al. reported that the basic variant, which was due to Hc-Asp-102 isomerization to isoaspartic acid in one of the heavy chains, has significantly reduced potency (Harris et al., 2001). Interestingly, the degree of both asparagine deamidation and aspartic acid isomerization in trastuzumab increases when incubated under physiological conditions (Schmid et al., 2018; Spanov et al., 2021). Earlier studies have shown that modifications in the CDRs of trastuzumab lead to a decrease in the affinity for the target receptor (Schmid et al., 2018; Dakshinamurthy et al., 2017). Here we wanted to investigate whether the formation of the complex influences the level of modifications or whether the modifications occur independently of the complex formation.

In the present study, the level of CDR modifications was assessed when trastuzumab was stressed under physiological conditions [phosphate-buffered saline (PBS), pH 7.4, and 37°C] free or in complex with HER2. Cation-exchange chromatography was used to isolate unmodified trastuzumab and size-exclusion chromatography (SEC) served to study trastuzumab–HER2 complex formation and stability. Peptide mapping by liquid chromatography-mass spectrometry (LC-MS) was used to localize modifications in the CDRs and to compare the relative level of modifications.

## Materials and Methods

### Materials and Chemicals

Trastuzumab (Herceptin®, Lot N3024H10) was obtained from Roche (Grenzach-Wyhlen, Germany). Human HER2/ErbB2 Protein (His Tag protein, extracellular domain Thr23–Thr652; cat # HE2-H5225) was purchased from Acrobiosystems (Delaware, United States). 2-(N-morpholino)ethanesulfonic acid, 4-morpholineethanesulfonic acid monohydrate (MES monohydrate, cat # 69892), 4-(2-hydroxyethyl)piperazine-1-ethanesulfonic acid (HEPES, cat # H4034), N,N-bis(2-hydroxyethyl)glycine (bicine, cat # B3876), 3-(cyclohexylamino)-2-hydroxy-1-propanesulfonic acid (CAPSO, cat # C2278), 3-(cyclohexylamino)-1-propanesulfonic acid (CAPS, cat # C6070), sodium chloride (cat # 746398), DL-dithiothreitol (DTT, cat # D0632), iodoacetamide (IAA, cat # 16125), and sodium deoxycholate (SDC, cat # 30970) were obtained from Sigma-Aldrich (St. Louis, Missouri, United States). PBS 10× (cat # 14200-067) was purchased from Thermo Fisher Scientific (Waltham, MA, United States). Trypsin/Lys-C mix, Mass Spec Grade (cat # V5073), was obtained from Promega (Madison, WI, United States). Difluoroacetic acid (DFA, cat # 162120025) was acquired from Acros Organics (Fair Lawn, NJ, United States).

### Cation-Exchange Chromatography

Cation-exchange chromatography was performed by pH-gradient separation on a MabPac SCX-10 (4 × 250 mm, 5 μm, Thermo Fisher Scientific, cat # 078655) column using an Agilent 1200 HPLC system as previously described (Spanov et al., 2021). pH gradient buffers were prepared according to Lingg et al. (2013), where buffer A (HEPES, Bicine, CAPSO, CAPS) had a pH of 8.0, and buffer B (Bicine, CAPSO, CAPS) had a pH of 10.5, respectively. A gradient change from 0% to 60% B over 10 column volumes (62.8 min) at a 0.5 ml/min flow rate was used for the separation of charge variants at room temperature. UV absorbance was measured at 280 nm with Agilent G4212B Diode Array Detector. The main peak (main fraction) from the cation-exchange chromatography was fractionated from non-stressed trastuzumab in several consecutive runs to obtain unmodified trastuzumab. The main fraction was collected into a Protein LoBind 96-well plate (cat # 0030504208; Eppendorf, Hamburg, Germany) filled with 100 µl of 300 mM MES buffer (pH 6) for pH neutralization to prevent deamidation. The main fraction was concentrated and buffer exchanged to 10 mM MES pH 6 with Amicon Ultra-2 Centrifugal Filter Units (cut-off 50 kDa, UFC205024, Merck Millipore, Darmstadt, Germany). Protein concentration of the main fraction was measured on a NanoPhotometer® N120 (Implen GmbH, Munich, Germany) at 280 nm (extinction coefficient was 1.36 L/g•cm).

### Trastuzumab–HER2 Complex Formation at Different Trastuzumab to HER2 Ratios

Trastuzumab–HER2 complexes at molar ratios of 1:1, 1:2, and 2:1 were made by mixing corresponding amounts of 0.5 μg/μl HER2 and 0.5 μg/μl of trastuzumab main fraction followed by incubation at room temperature for 2 h at 500 rpm. The molecular weight of the HER2 extracellular domain (ECD) 115 kDa, determined by sodium dodecyl sulfate polyacrylamide gel electrophoresis (SDS-PAGE) (Supplementary Figure S1), was used to make complexes with different molar ratios. Samples were analyzed by SEC.

### Size-Exclusion Chromatography

An Agilent 1200 HPLC system coupled with a TSKgel SuperSW mAb HR (7.8 mm × 300 mm, 4 μm, Tosoh Bioscience, United States) column was used to study formation and stability of the trastuzumab–HER2 complex. The mobile phase was 50 mM phosphate buffer pH 6.5 containing 450 mM NaCl. Isocratic elution at a flow rate of 1 ml/min at room temperature was performed, and eluting proteins were detected by UV absorbance at 280 nm.

### Stressing Free Trastuzumab and the Trastuzumab–HER2 Complex

About 5.65 µg of unmodified trastuzumab (main fraction) in 20 µl PBS was stressed free and in complex with 8.7 µg of HER2 (trastuzumab to HER2 ratio 1:2) at 37°C for up to 4 weeks. Samples containing trastuzumab and HER2 were first left at room temperature for 2 h for complex formation and then incubated at 37°C for the indicated time periods. 5 µl of the stressed sample was injected into the size-exclusion column.

### LC-MS/MS Peptide Mapping

Stressed samples were denatured and reduced in the presence of 0.5% SDC in 50 mM HEPES buffer pH 7 and 5 mM DTT at 60°C for 30 min. Alkylation was performed by adding IAA to a final concentration of 15 mM at room temperature for 20 min in the dark. Subsequently, a trypsin/Lys-C mix was added to the samples at a ratio of 25:1 (protein to enzyme), and proteins were digested for 6 h at 37°C. SDC was removed by precipitation prior to LC-MS analysis by adding 0.2% final concentration of DFA and centrifugation at 10,000 rpm for 10 min.

Digested samples were loaded on an Acclaim® PepMap® RSLC column (0.3 × 150 mm, 2 μm, 100 Å, Thermo Fisher Scientific) for chromatographic separation using a gradient from 2% to 35% B in 65 min, where mobile phase A consisted of 0.1% formic acid in water and mobile phase B of 0.1% formic acid in acetonitrile. The flow rate was 5 μl/min, and the column temperature was set to 40°C. LC-MS/MS peptide mapping analysis was conducted using an Eksigent NanoLC 425 system with a microflow pump (1–10 µl) coupled to a SCIEX TT6600 quadrupole-time-of-flight (QTOF) mass spectrometer with an OptiFlow® source (SCIEX, Toronto, Canada). The following source settings were applied: ion source gas 1 (GS1) 10 psi, ion source gas 2 (GS2) 20 psi, curtain gas (CUR) 25 psi, temperature (TEM) 100°C, IonSpray Voltage Floating (ISVF) 4.5 kV, and declustering potential (DP) 90 V. Measurements were performed in the data-dependent acquisition mode where one MS scan was followed by MS/MS of the top five most intense precursor ions. The MS scan range was 350–2m000 m/z. The threshold for precursor ion selection was set at 500 counts per second, and the charge states for the MS/MS fragmentation of precursor ions were set to +2 to +5 with an exclusion window of 4 s after two occurrences.

BPV Flex 2.1 and PeakView (SCIEX, Toronto, Canada) were employed for data analysis. Precursor mass tolerance was set to 20 ppm, and fragment mass tolerance was set to 0.03 Da. Carbamidomethylation was defined as fixed modification, while methionine oxidation and asparagine deamidation were defined as variable modifications.

## Results and Discussion

### Isolation of Main Fraction of Trastuzumab by Cation-Exchange Chromatography

Trastuzumab is known to be heterogeneous in terms of charge. In our previous study (Spanov et al., 2021) we have shown that cation-exchange chromatography is an appropriate tool to study the stability of trastuzumab under stress conditions. Stressing trastuzumab for up to 3 weeks under physiological conditions (PBS buffer pH 7.4 and 37°C) resulted in complex chromatographic profiles. The source of charge heterogeneity was due to Lc-Asn-30, Hc-Asn-55, and Hc-Asn-387 deamidation, Hc-Asp-102 isomerization, and N-terminal pyroglutamate formation at the heavy chain. To initiate our study with unmodified trastuzumab, we decided to work with the main fraction after isolation by cation-exchange chromatography to discriminate modifications that occurred during stressing from those that were already present in clinical grade trastuzumab.

### Size-Exclusion Chromatography to Study Formation of the Trastuzumab–HER2 Complex

**FIGURE 1:**
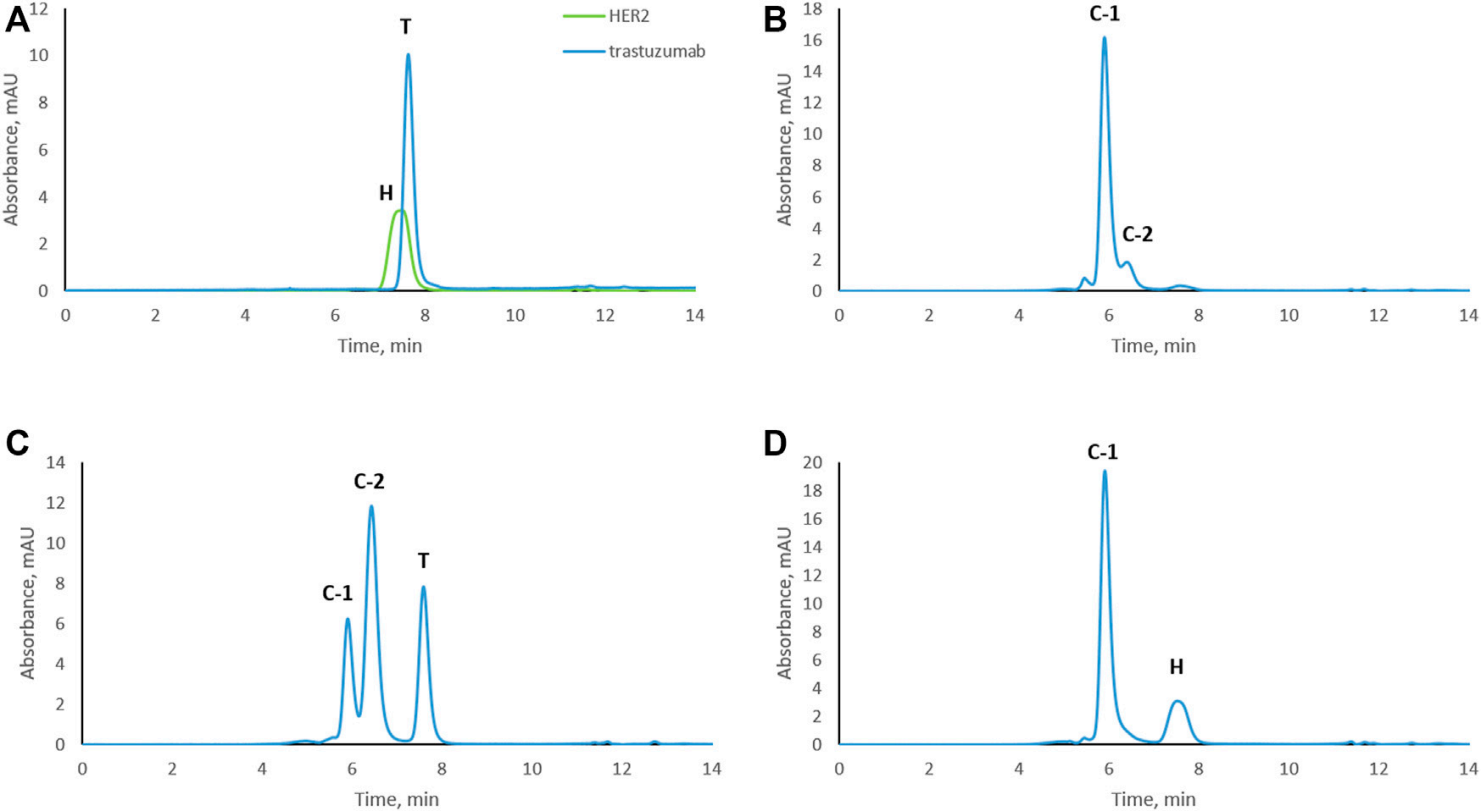
Size-exclusion chromatograms of trastuzumab–HER2 complexes formed at different molar ratios. **(A)** Overlay chromatograms of trastuzumab and HER2. **(B)** Complex formed at 1:1 molar ratio. **(C)** Complex formed at 2:1 molar ratio (excess of trastuzumab). **(D)** Complex formed at 1:2 molar ratio (excess of HER2). C-1, complex-1 at 5.9 min; C-2, complex-2 at 6.4 min; T, trastuzumab at 7.7 min; H, HER2 at 7.5 min. UV absorbance was measured at 280 nm.

To study trastuzumab–HER2 complex formation at different molar ratios, samples were subjected to size exclusion chromatography. Trastuzumab and HER2 were also analyzed separately as controls. An overlay of the chromatograms of trastuzumab and HER2 are shown in Figure 1A. Interestingly, HER2 ECD eluted slightly earlier than trastuzumab which has a molecular weight of around 149 kDa. The calculated molecular weight of HER2 ECD based on the amino acid sequence was 70.2 kDa. The molecular weight of HER2 ECD determined by SDS-PAGE was around 115 kDa (Supplementary Figure S1), and this molecular weight was used to prepare complexes at different ratios (as mentioned in the *Materials and Methods* section). However, additional molecular weight measurements by mass photometry (Refeyn Ltd., Oxford, England) and matrix-assisted laser desorption/ionization time-of-flight mass spectrometry (MALDI-TOF MS) (Bruker Daltonics, Bremen, Germany) showed the value lower than determined by SDS-PAGE, in the range of 80–90 kDa showing high heterogeneity of the protein, probably due to glycosylation (Supplementary Figures S2, S3). Despite the discrepancies in molecular weight values determined by different techniques, none of the values were higher than 149 kDa. Since HER2 ECD eluted earlier than trastuzumab in SEC, we assume that HER2 ECD might be in dimer form in solution. A sample in which the complex was formed at a 1:1 molar ratio resulted in one main peak [complex-1 (C-1) at 5.9 min] and a shoulder peak (C-2) at 6.4 min (Figure 1B) next to a very minor, earlier eluting peak. Complex formation at a trastuzumab to HER2 ratio of 2:1 showed free trastuzumab, complex C-2 as the major peak, and complex C-1 (Figure 1C) indicating that C-2 represents a 1:1 complex while C-1 contains two molecules of HER2 for each molecule of trastuzumab. Complex formation in the presence of an excess of HER2 (trastuzumab: HER2 1:2) resulted in complex C-1 and a peak for free HER2 (Figure 1D) but no remaining free trastuzumab. Conditions under which there was no remaining free trastuzumab (1:2 trastuzumab: HER2 molar ratio) were chosen for the subsequent stress tests to assure that modifications occurred while trastuzumab was in complex with HER2.

### Stressing the Trastuzumab–HER2 Complex

**FIGURE 2:**
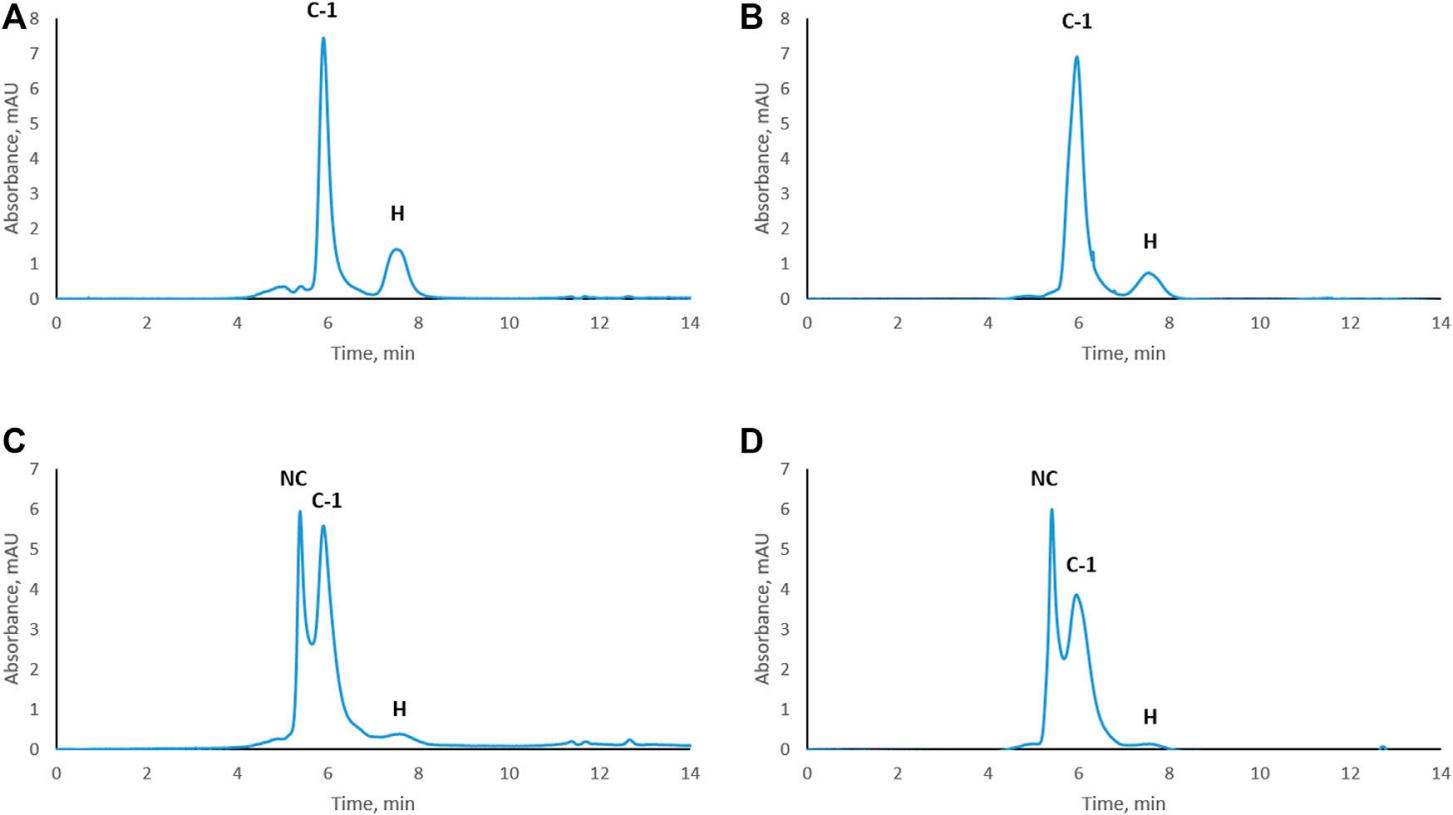
Size-exclusion chromatograms of the trastuzumab–HER2 complex stressed under physiological conditions. Complex stressed for 1 week **(A)**, 2 weeks **(B)**, 3 weeks **(C)**, and 4 weeks **(D)**. NC, new complex; C-1, complex-1; H, HER2. UV absorbance was measured at 280 nm.

**FIGURE 3:**
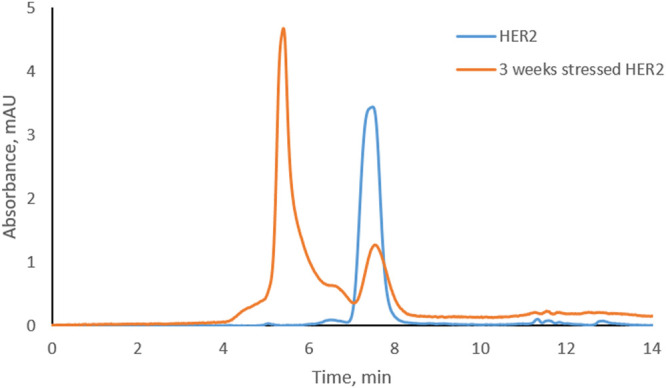
Overlay of size-exclusion chromatograms of free HER2 at the beginning and after 3 weeks under physiological conditions.

Trastuzumab was stressed free and in complex with HER2 (trastuzumab: HER2 molar ratio 1:2, complex C-1; see Figure 1D) under physiological conditions. Aliquots were taken every week to study the level of CDR modifications. Size exclusion chromatography was used to assure that the complex did not dissociate under these conditions and that there was no free trastuzumab in the solution. Figure 2 shows the chromatographic profiles after 1, 2, 3, and 4 weeks of stressing. There was no change from the initial profile (see Figure 1D) after 1 or 2 weeks (Figures 2A, B) with complex C-1 remaining the main peak and free HER2 being still present. It must be noted though that the peak for the C-1 complex broadened slightly after 2 weeks and the peak for free HER2 diminished. A major change was observed after 3 weeks when a new, earlier eluting complex appeared (NC, retention time of 5.4 min) and the excess of free HER2 had almost disappeared. (Figures 2C, D). The broadening of peak C-1 after 2 weeks may have been due to the formation of a minor amount of NC, which was not separated from the excess of C-1. We assume that the new complex is due to oligomerization of HER2. To check whether this is the case, free HER2 was stressed for 3 weeks under the same conditions showing that HER2 oligomerizes to a form eluting at about 5.4 min in the absence of trastuzumab (Figure 3). This indicates that the newly observed complex after 3 weeks is due to HER2–HER2 interactions.

Based on SEC results, we assume that C-1 is a complex between a HER2 dimer and trastuzumab. HER2 is known to form homo- and heterodimers through sub-domain II (Adams et al., 2006; Smith et al., 2012). However, trastuzumab binds to sub-domain IV, which should still be available for trastuzumab binding even if HER2 is present as a dimer. C-2 is likely a 1:1 complex between a HER2 monomer and trastuzumab, since it was the main complex formed when trastuzumab was in excess. The presence of free HER2-ECD in Figure 1D can be explained by the use of a molecular weight of 115 kDa as determined by SDS-PAGE for the preparation of complexes. Upon detection of free HER2-ECD, we reassessed the molecular weight by mass photometry and MALDI-TOF MS and found that it was approximately 85 kDa. Considering this molecular weight, the actual trastuzumab:HER2 ratio in Figure 1D is calculated to be approximately 1:2.7 rather than 1:2, which may explain the free excess of HER2-ECD. It is important to note that we had a stochiometric amount of HER2-ECD or an excess to assure that there was no free trastuzumab during the stress study. In addition, having free HER2 also allowed to observe formation of higher-order complexes after 3 and 4 weeks of stressing trastuzumab–HER2 complex (Figures 2C, D) and also to find out that HER2 can oligomerize in solution upon stressing (Figure 3).

### Peptide Mapping of Trastuzumab Stressed Free and in Complex With HER2

**FIGURE 4:**
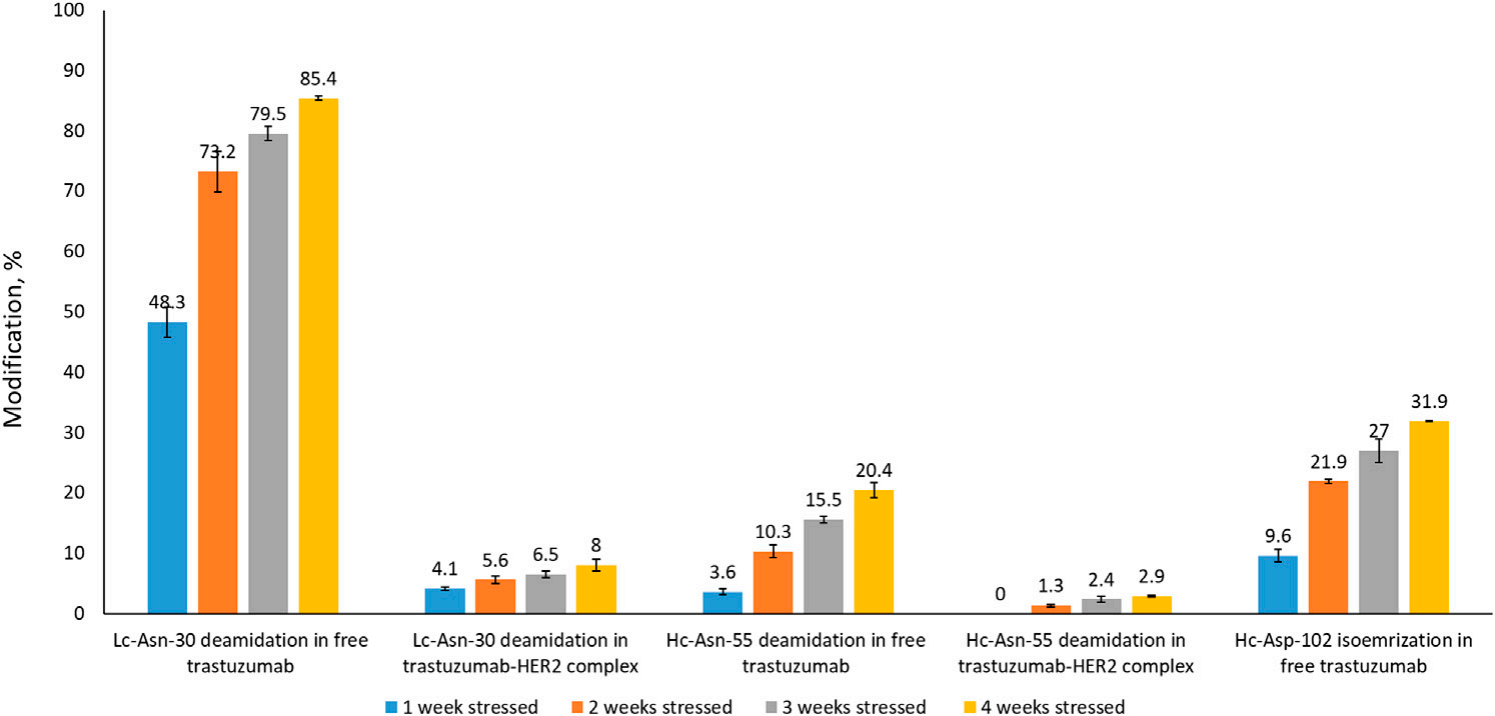
Peptide mapping results of trastuzumab stressed free and in complex with HER2 for 1, 2, 3, or 4 weeks. Isomerization of Hc-Asp-102 was not detected in the trastuzumab–HER2 complex. Numbers given here are an average of two measurements.

In order to assess whether complex formation with HER2 affects critical modifications in the CDRs of trastuzumab, we analyzed trastuzumab after stressing for up to 4 weeks by LC-MS-based peptide mapping. Native and deamidated peptides were distinguished by being chromatographically separated and due to differences in their MS/MS spectra (Supplementary Figures S4−S9). Asp isomerization resulted in the formation of isobaric peptides which could not be distinguished based on their MS or MS/MS spectra. However, chromatographic separation between the Asp and isoAsp forms was achieved. Deamidation of Lc-Asn-30 located in CDR1 was reported previously as a position that is highly susceptible to deamidation (Harris et al., 2001; Schmid et al., 2018; Spanov et al., 2021). Our peptide mapping results of free trastuzumab agree with these observations showing up to 85% deamidation after 4 weeks of stressing (Figure 4). However, when trastuzumab was stressed for 4 weeks in complex with HER2, Lc-Asn-30 deamidation was decreased to about 8%. Hc-Asp-102 isomerization, a modification that critically affects activity, increased to 32% after 4 weeks when trastuzumab was stressed in its free form. Strikingly, isomerization was not detected at all when trastuzumab was stressed in complex with HER2. Hc-Asn-55 is another position that is known to deamidate albeit at a slower rate compared to Lc-Asn-30. While 20% deamidation of Hc-Asn-55 was detected when free trastuzumab was stressed for 4 weeks, this level decreased to about 3% in the trastuzumab–HER2 complex.

Deamidation is known to be sequence dependent, and usually motifs such as NG and NS may have higher deamidation rate. However, this is not always the case as it depends also on conformational flexibility and solvent exposure of the particular position (Lu et al., 2018). In the case of trastuzumab, deamidation of N30 in the light chain, which is followed by threonine (T), is faster compared to the deamidation of N55 in the heavy chain, which is followed by glycine (G). These differences in deamidation of N residues in trastuzumab have been reported in the literature, and our results are in agreement with previously published results (Harris et al., 2001; Schmid et al., 2018; Spanov et al., 2021).

Peptide mapping is a widely used approach for the characterization of mAbs in terms of confirming their primary amino acid sequence as well as to localize potential modifications. The results presented here are based on label-free analyses, where the level of modification is calculated based on the ratio of the chromatographic peak area of the modified peptide to the sum of the peak areas of the modified and unmodified peptides. As this approach does not take differences in ionization efficiency between non-modified and modified peptides into account, it must be considered semi-quantitative. However, considering the large differences in deamidation or isomerization of trastuzumab free in solution and in complex with HER2, it is safe to assume that complex formation reduces the level of modification of these amino acid residues to a very significant extent.

Early studies on trastuzumab humanization showed that both Lc-Asn-30 and Hc-Asp-102 are important for antigen binding (Kelley and O’Connell, 1993; Gerstner et al., 2002). The fact that Lc-Asn-30 and Hc-Asn-55 were able to deamidate when trastuzumab was stressed in complex with HER2 indicates that there is still some conformational flexibility around these residues in the complex, while isomerization of Hc-Asp-102 was not detected at all when trastuzumab was stressed when bound to HER2. Isomerization of an aspartic acid residue introduces a major conformational change in the peptide backbone through the introduction of an extra methylene group (Du et al., 2012; Liu et al., 2014). Our results indicate that there is no flexibility around Hc-Asp-102 to accommodate such a conformational change when trastuzumab is in a complex with HER2. This may be due to the tight binding of CDR3 to HER2, as indicated by the previously reported importance of heavy chain CDR3 for antigen binding (Xu and Davis, 2000; D’Angelo et al., 2018). In fact, Shang et al. were able to prepare an alternative affinity binder for HER2 by mimicry of CDR3 (Shang et al., 2012). Moon et al. showed that substitution of a few amino acid residues in CDR3 of trastuzumab, including Hc-Asp-102, for other residues may increase the binding affinity of trastuzumab to HER2 (Moon et al., 2016). In their original study, Harris et al. reported that isomerization of Hc-Asp-102 reduces the potency of trastuzumab to block proliferation of a breast cancer cell line significantly (Harris et al., 2001). The absence of isomerization of Hc-Asp-102 in CDR3 when trastuzumab was stressed in complex with HER2 may be taken as indirect indication of the tight interaction between CDR3 of trastuzumab and HER2.

## Conclusion

This study investigated the effect of complex formation on modifications in three different positions of CDRs in the therapeutic mAb trastuzumab when stressed under physiological conditions. Formation of the trastuzumab–HER2 complex and its stability under stress conditions was followed by SEC. Analysis by size exclusion chromatography indicated further that there are complexes with different stoichiometries, the 1:2 complex (trastuzumab: HER2) being the most abundant for up to 2–3 weeks of incubation, after which HER dimerization leads to formation of a larger, earlier eluting complex. Complex formation has a major effect on the deamidation of Lc-Asn-30 and Hc-Asn-55 but did not prevent it altogether. However, complex formation prevented isomerization of Hc-Asp-102, which was undetectable.

Further structural studies are needed to elucidate the underlying reasons for our observations. *In vivo* complex formation may explain some of the puzzling observations of different levels of deamidation of Hc-Asn-55 in breast cancer patients treated with trastuzumab (Bults et al., 2016). Since the rate of the deamidation reaction is not known to be affected by enzymatic activities or other biochemical factors other than pH and temperature, it is fair to hypothesize that the different levels in patients may be due to different amounts of trastuzumab being complexed with HER2. This hypothesis is intriguing but must await further testing before any conclusions can be drawn.

## Data Availability

The raw data supporting the conclusion of this article will be made available by the authors, without undue reservation.
