# Supplementary figures and images for: Effect of Trastuzumab–HER2 Complex Formation on Stress-Induced Modifications in the CDRs of Trastuzumab
Source: Front Chem. 2022 Jan 3;9:794247. doi: 10.3389/fchem.2021.794247 (PMC8762049; doi:10.3389/fchem.2021.794247)

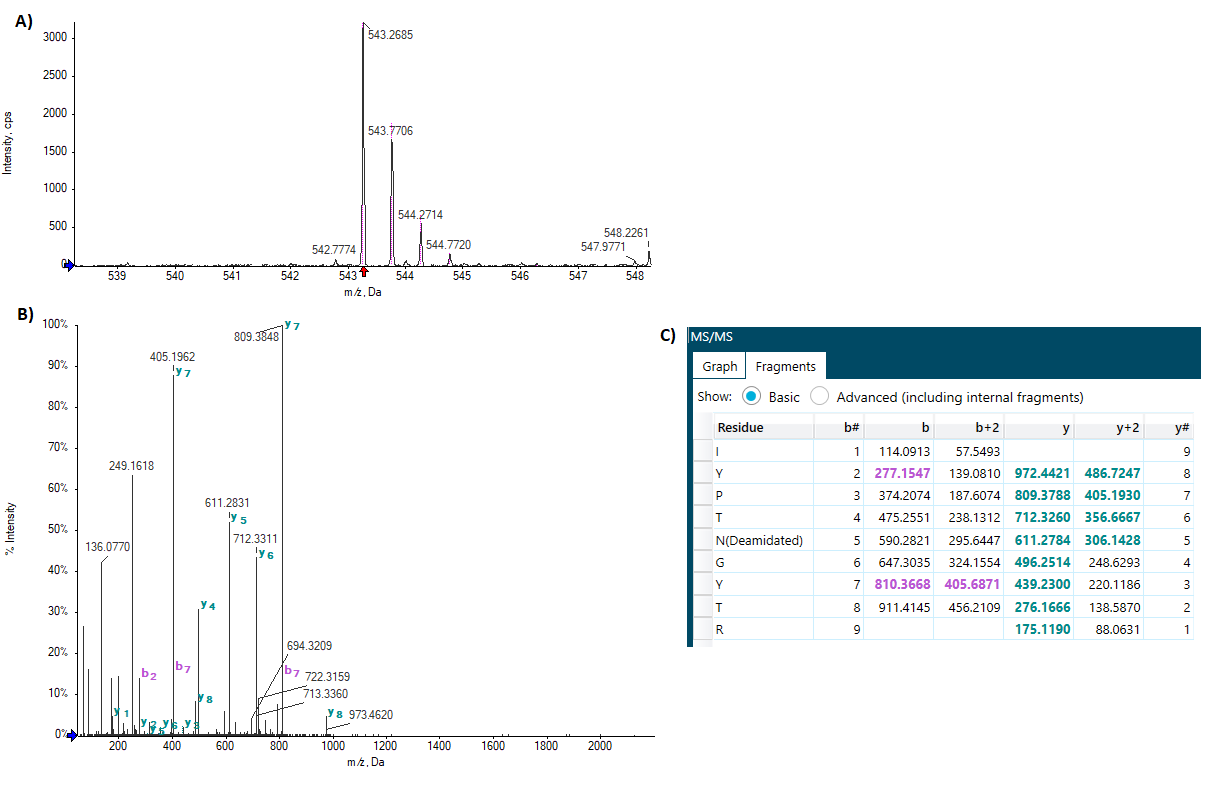

Supplement: Supplementary file 1 [file Image9.TIFF]

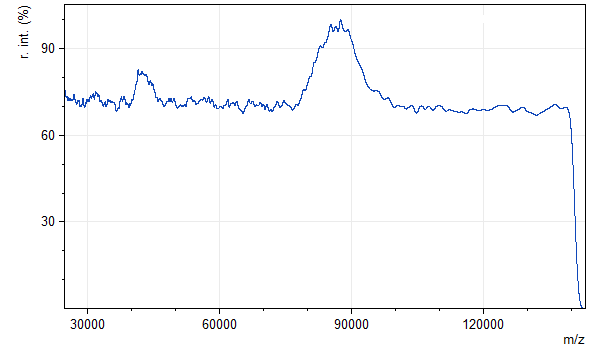

Supplement: Supplementary file 2 [file Image3.TIF]

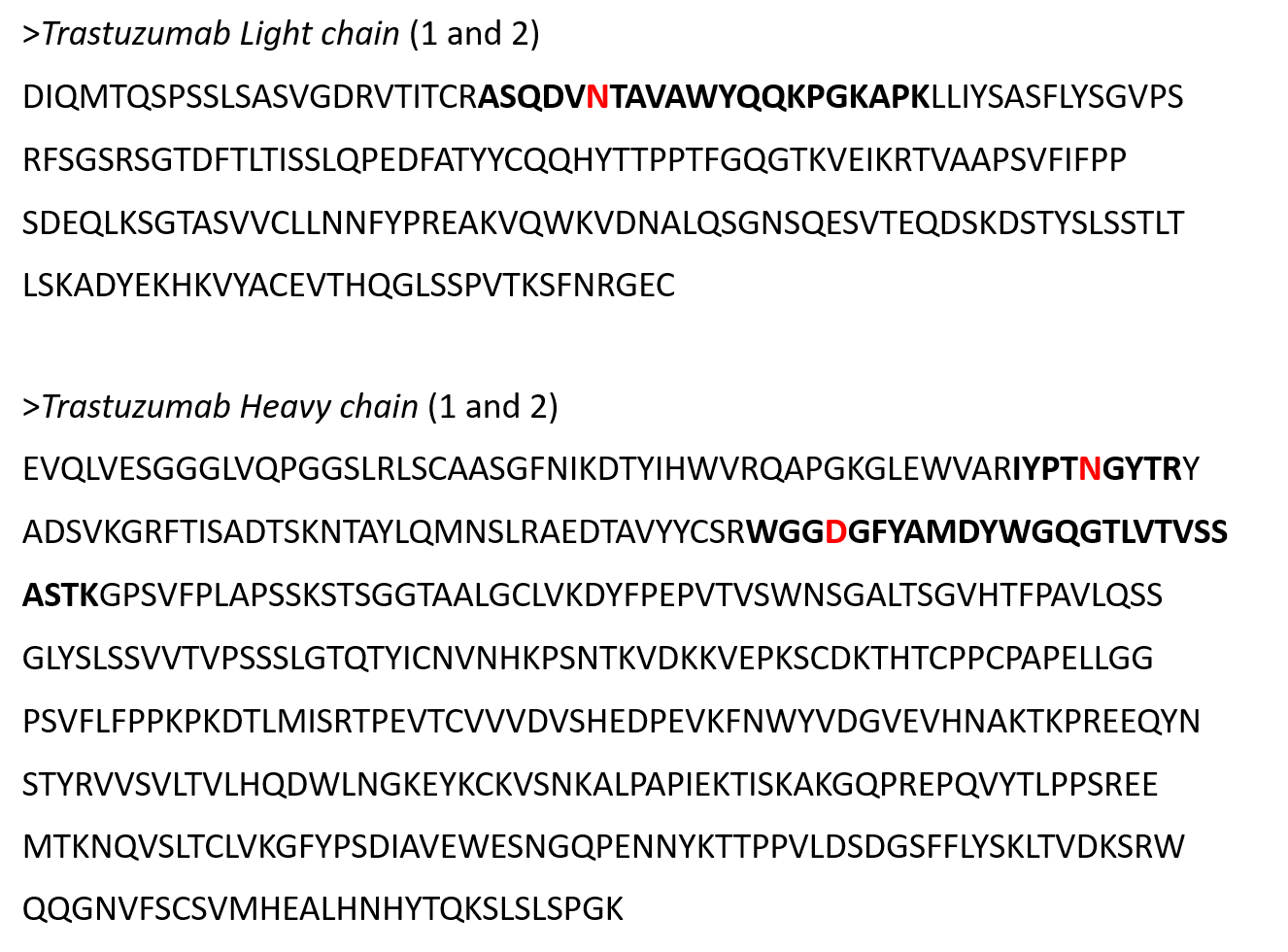

Supplement: Supplementary file 3 [file Image4.TIF]

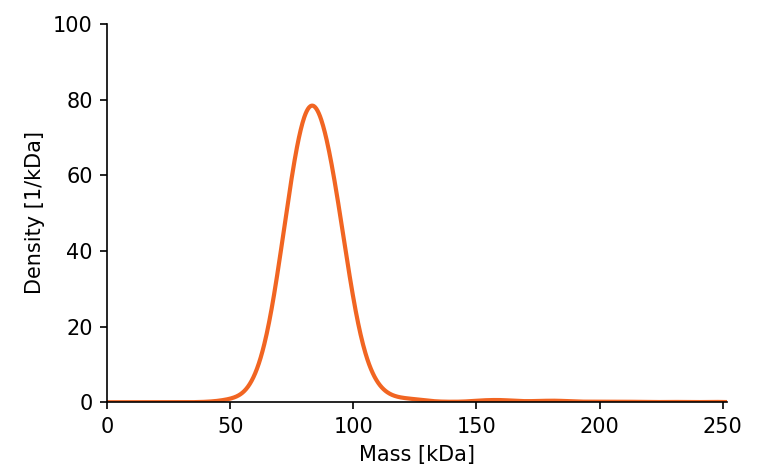

Supplement: Supplementary file 4 [file Image2.TIF]

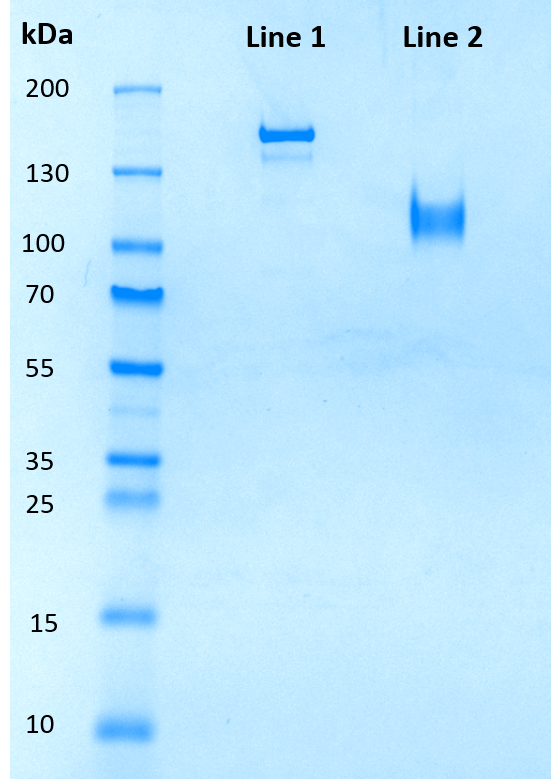

Supplement: Supplementary file 5 [file Image1.TIF]

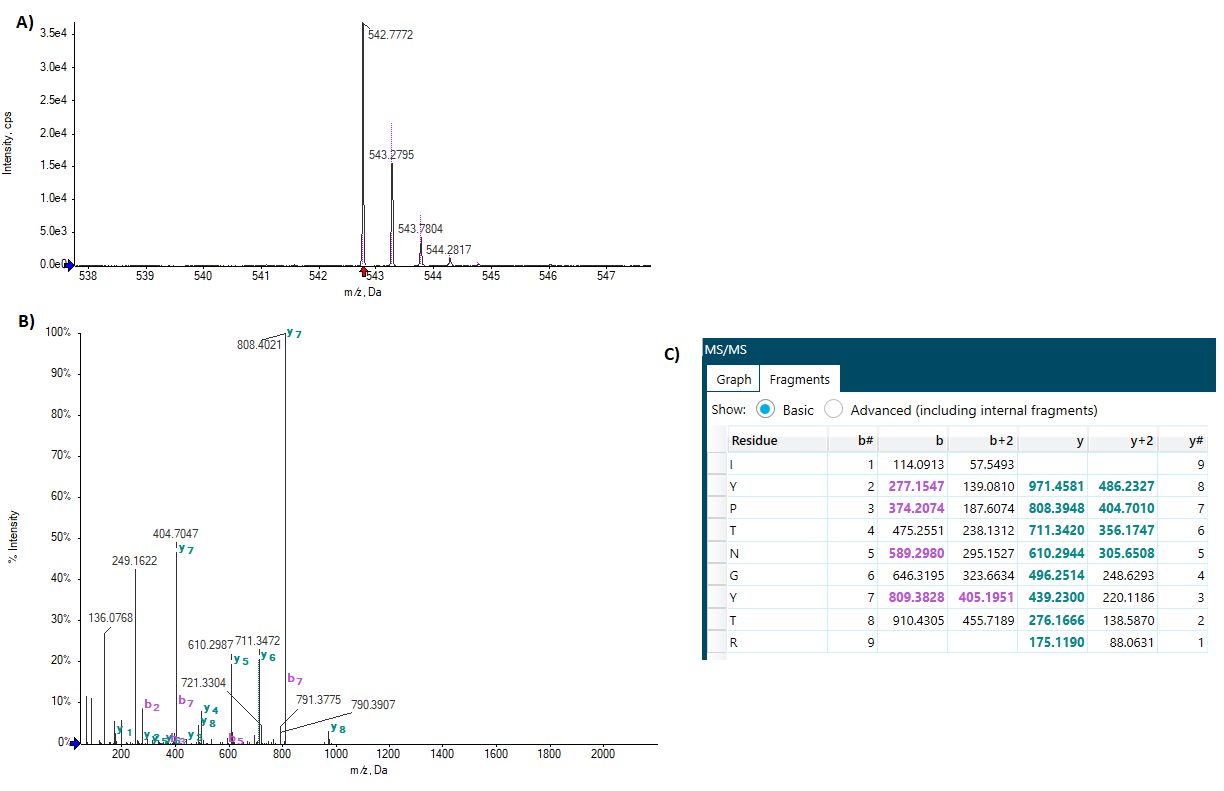

Supplement: Supplementary file 6 [file Image8.TIFF]

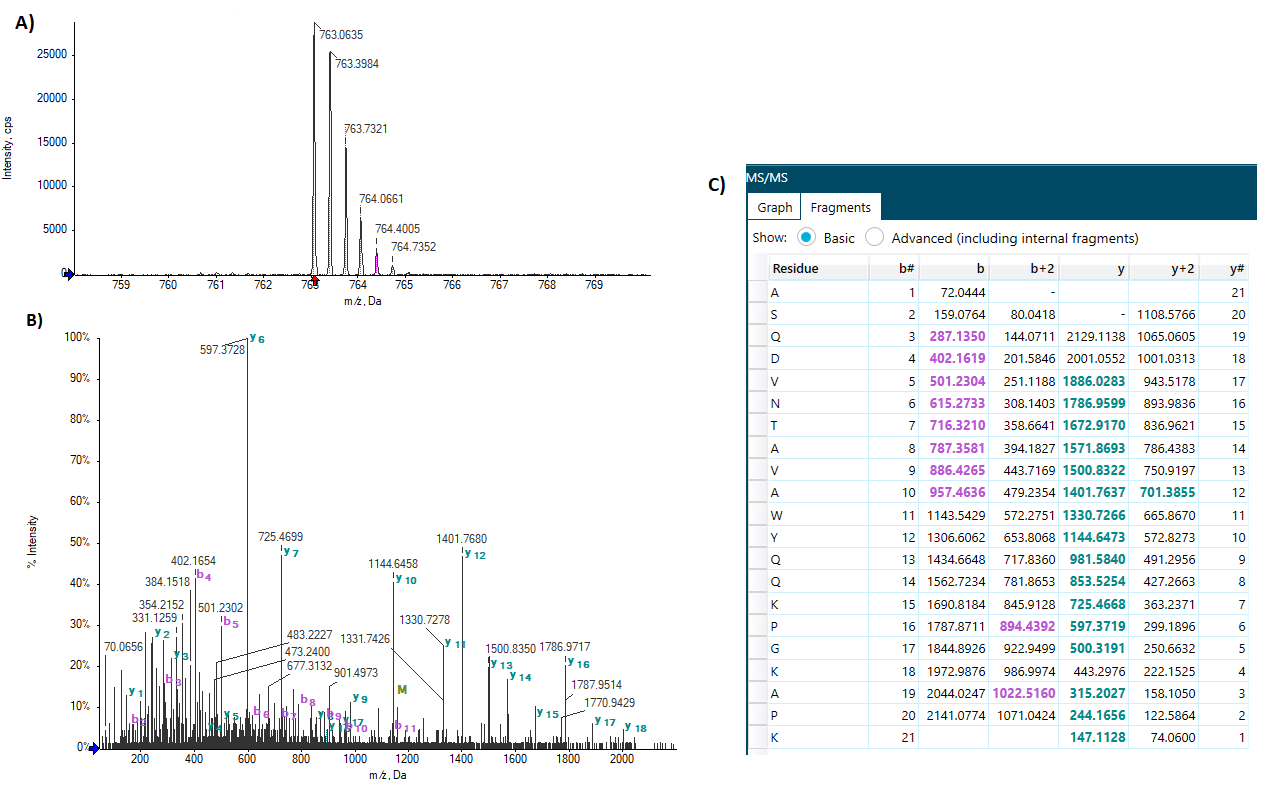

Supplement: Supplementary file 7 [file Image6.TIFF]

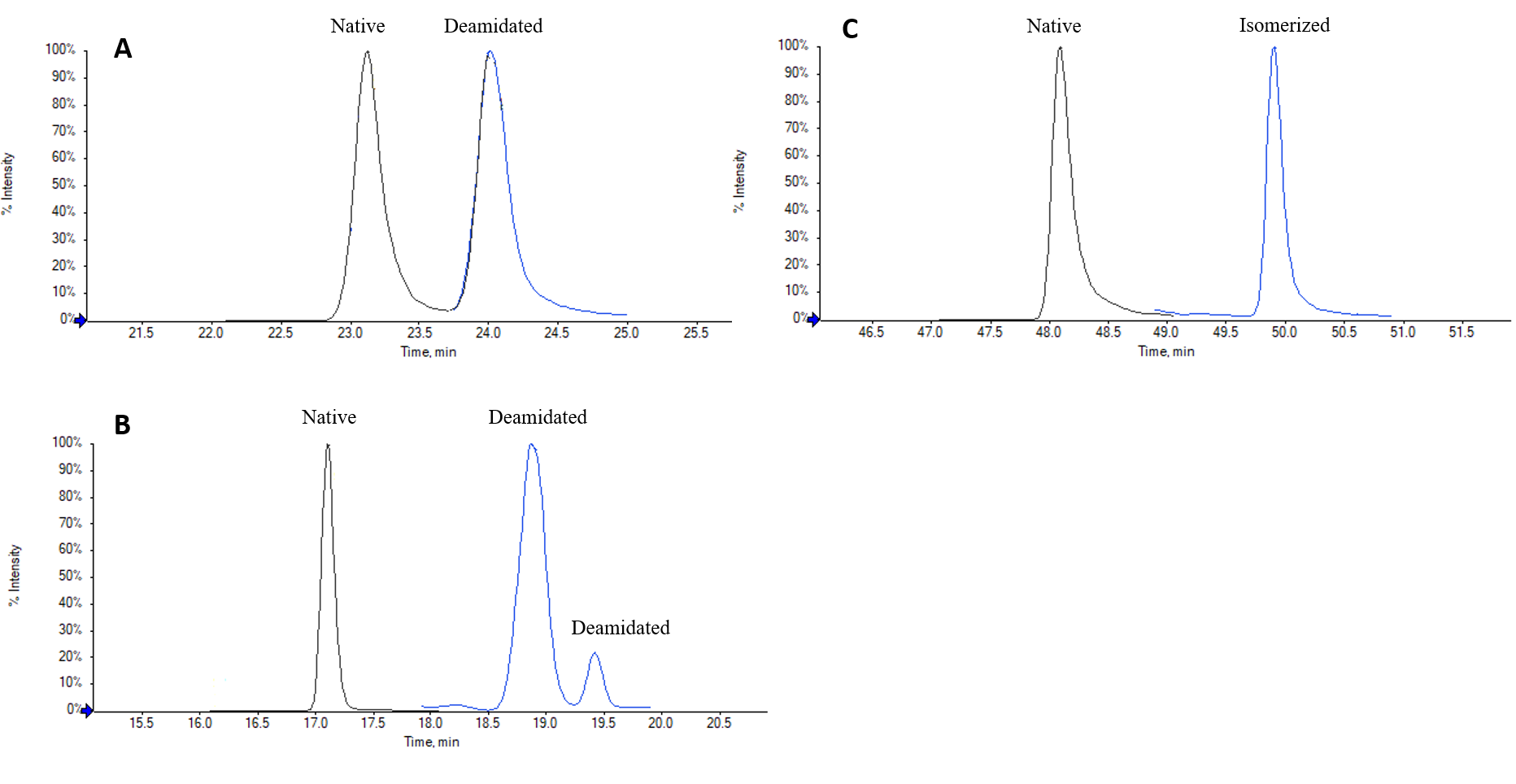

Supplement: Supplementary file 8 [file Image5.TIF]

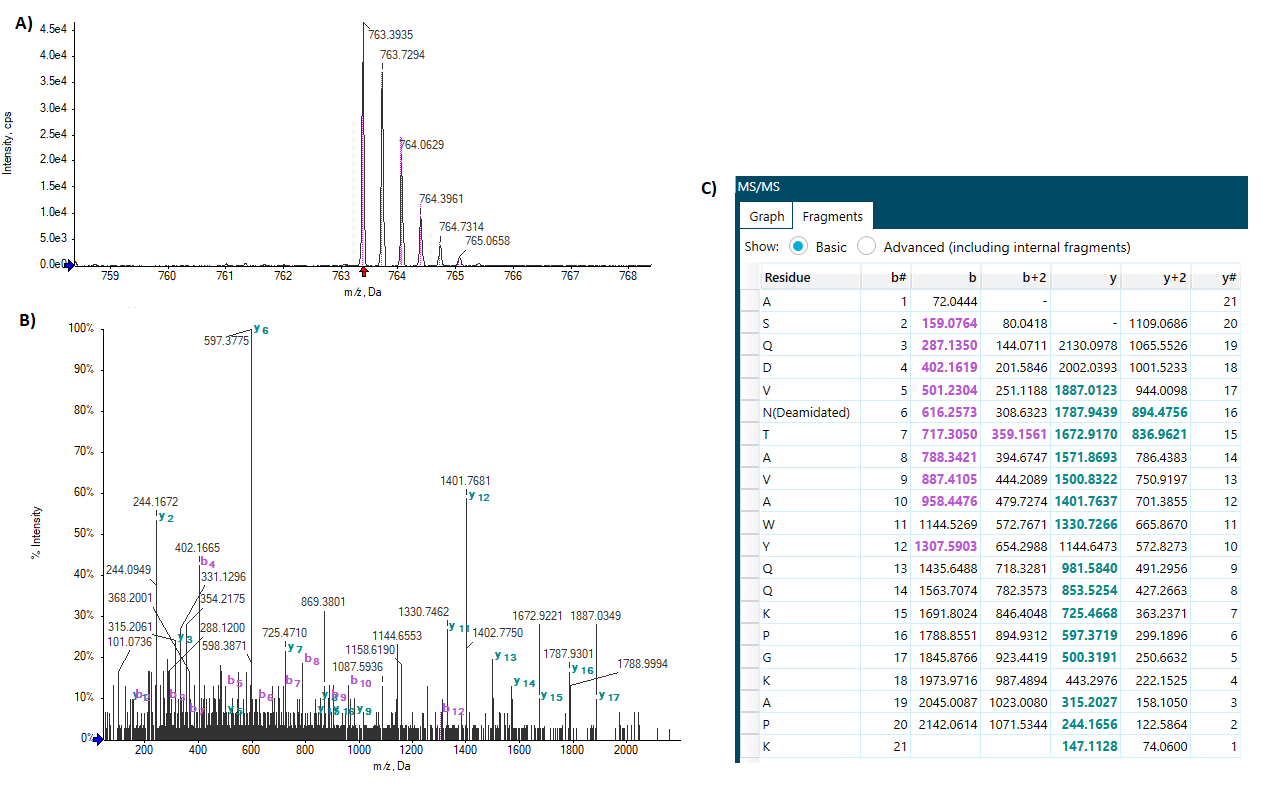

Supplement: Supplementary file 9 [file Image7.TIFF]
